# Zeb2 Regulates Myogenic Differentiation in Pluripotent Stem Cells

**DOI:** 10.3390/ijms21072525

**Published:** 2020-04-05

**Authors:** Ester Sara Di Filippo, Domiziana Costamagna, Giorgia Giacomazzi, Álvaro Cortés-Calabuig, Agata Stryjewska, Danny Huylebroeck, Stefania Fulle, Maurilio Sampaolesi

**Affiliations:** 1 Department of Neuroscience Imaging and Clinical Sciences, University “G. d’Annunzio” of Chieti-Pescara, 66100 Chieti, Italy; es.difilippo@unich.it (E.S.D.F.); stefania.fulle@unich.it (S.F.); 2 Department of Development and Regeneration, KU Leuven, 3000 Leuven, Belgium; domiziana.costamagna@kuleuven.be (D.C.); giorgia.giacomazzi@histogenex.com (G.G.); a.stryjewska@ucl.ac.uk (A.S.); d.huylebroeck@erasmusmc.nl (D.H.); 3 Genomics Core Leuven, Centre for Human Genetics KU Leuven, 3000 Leuven, Belgium; alvaro.cortes@uzleuven.be; 4 Department of Cell Biology, Erasmus University Medical Center, 3015 CN Rotterdam, The Netherlands; 5 Human Anatomy Unit, Department of Public Health, Experimental and Forensic Medicine, University of Pavia, 27100 Pavia, Italy

**Keywords:** pluripotent stem cells, myogenic progenitors, Zinc finger E-box-binding homeobox 2, myogenic differentiation, skeletal muscle, signalling pathways, myogenesis

## Abstract

Skeletal muscle differentiation is triggered by a unique family of myogenic basic helix-loop-helix transcription factors, including MyoD, MRF-4, Myf-5, and Myogenin. These transcription factors bind promoters and distant regulatory regions, including E-box elements, of genes whose expression is restricted to muscle cells. Other E-box binding zinc finger proteins target the same DNA response elements, however, their function in muscle development and regeneration is still unknown. Here, we show that the transcription factor zinc finger E-box-binding homeobox 2 (Zeb2, Sip-1, Zfhx1b) is present in skeletal muscle tissues. We investigate the role of Zeb2 in skeletal muscle differentiation using genetic tools and transgenic mouse embryonic stem cells, together with single-cell RNA-sequencing and in vivo muscle engraftment capability. We show that Zeb2 over-expression has a positive impact on skeletal muscle differentiation in pluripotent stem cells and adult myogenic progenitors. We therefore propose that Zeb2 is a novel myogenic regulator and a possible target for improving skeletal muscle regeneration. The non-neural roles of Zeb2 are poorly understood.

## 1. Introduction

Zeb2 is a two-handed zinc-finger/homeodamin protein closely related to Zeb1 (δEF1, Zfhx1a) [1]. Both factors regulate gene transcription through similar, E-box-mediated DNA-binding [2,3]. However, in vivo, Zeb1 and Zeb2 are functionally distinct in many contexts, exhibiting strikingly different and only sometimes overlapping expression patterns [4,5]. Zeb2 is a strong binder of activated Smads, making it a nuclear fine-tuner of the transcriptional response to TGFβ/Nodal-Activin and BMP stimulation of cells [1,6,7,8]. This role has been shown to result, depending on cell stage/type, in the generation of not only anti-BMP, but also anti-Wnt, anti-Notch, and anti-Sox2 activities in embryonic development and cell differentiation/maturation, best documented in embryonic myelinogenesis, and adult Schwann cell differentiation and remyelination [9,10,11]. Heterozygous *ZEB2* mutations in humans cause Mowat-Wilson Syndrome (OMIM #235730), involving severe intellectual disability, Hirschsprung disease, epilepsy, and other developmental defects, with some patients also presenting with musculoskeletal anomalies [8,12,13].

*Zeb2* genetic inactivation in mouse embryonic stem cells (mESCs) causes defects in their pluripotency exit, making them stall as epiblast-like stem cells, and therefore compromising their neural and general differentiation [14]. 

It is well known that skeletal muscle development and differentiation are regulated by myogenic basic helix-loop-helix (bHLH) proteins. Similarly, several zinc-finger proteins have been described as regulators of muscle development and specific muscle gene expression. In this context, zinc-finger repressors of transcription could compete with myogenic bHLH proteins in regulating muscle differentiation processes. In fact, it has been reported that the overexpression of LIM/double zinc-finger protein promotes myogenic differentiation in the cell line C2C12 [15]. Zeb1 and Zeb2 have been proposed as candidate regulators and/or such competitors, based on biochemical analysis [2,16] and on phenotypes within embryonic somites in the respective knockout and compound mutant embryos and adult mice [4,17]. More recently, the presence of PW1 zinc-finger protein was reported in interstitial myogenic progenitors, and it is also required for migration ability of murine and human mesoangioblasts [18,19]. 

Here, we evaluated the myogenic potential of *Zeb2-null* and *R26_Zeb2* mESCs by single-cell RNA-sequencing, and tested muscle engraftment capability of the respective myogenic progenitors. *Zeb2* transgene (cDNA)-based expression was shown to impact positively on the myogenic differentiation potential of pluripotent stem cells and myogenic progenitors, identifying Zeb2 as a critical modulator of skeletal muscle differentiation.

Strikingly, we propose, for the first time, the function of Zeb2 in skeletal muscle differentiation. Our hypothesis is that Zeb2 has a crucial role in triggering myogenic differentiation. We also evaluated the complexity of the myogenic transcriptional regulation, including the TGFβ/BMP system, involved in myogenic commitment and differentiation. 

## 2. Results

### 2.1. The Upregulation of Zeb2 Positively Affects Myogenic Markers in mESCs Subjected to Skeletal Muscle Differentiation

**Figure 1:**
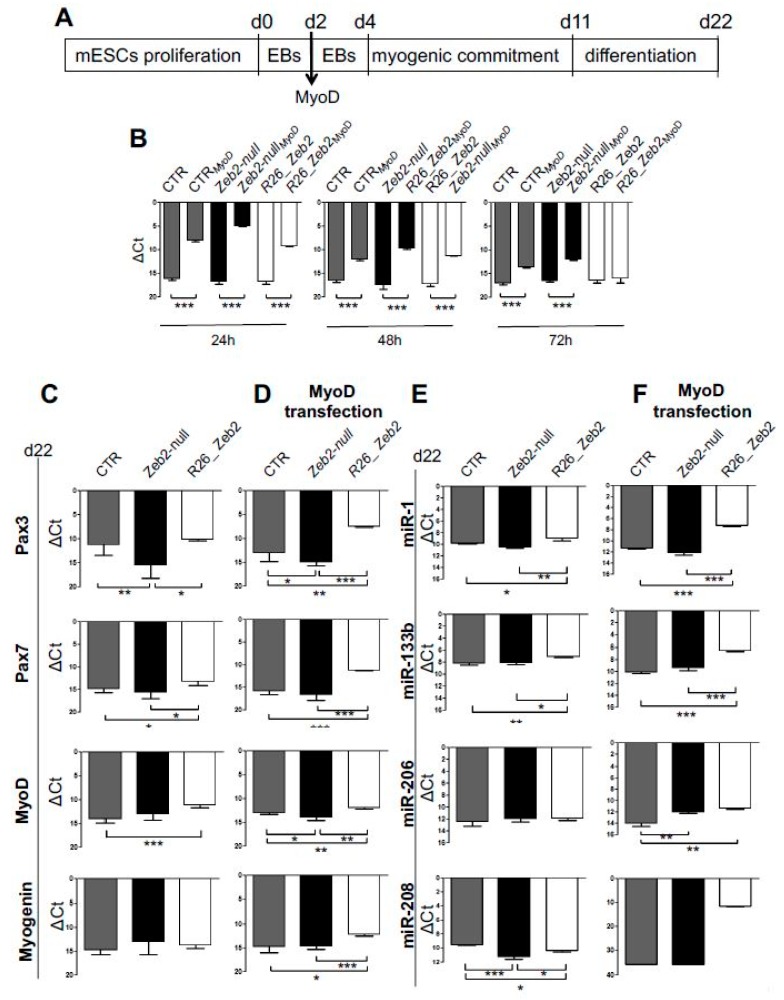
Muscle gene expression and MyomiR profile in *Zeb2-null* and *R26_Zeb2* mESCs subjected to myogenic differentiation. (**A**) Schematic representation of myogenic induction for mESCs. (**B**) The qRT-PCR analysis confirms the successful *MyoD* transient transfection in CTR, *Zeb2-null* and *R26_Zeb2* mESCs. At 24, 48, and 72 h from transfection, data are represented as ΔC*_t_*, normalized for the housekeeping *Gapdh*. Values are shown as mean ± s.d.; *n* = 3, *** *p* < 0.0001. (**C**,**D**) qRT-PCR analysis for skeletal muscle genes (*Pax3*, *Pax7*, *MyoD*, *Myogenin*) in CTR, *Zeb2-null* and *R26_Zeb2* mESCs, on day 22 from myogenic induction (**C**) or upon MyoD transfection (**D**). Each data point is represented as ΔC*_t_*, normalized for the housekeeping *Gapdh*. (**E**,**F**) qRT-PCR analysis for MyomiRs (miR-1, miR-133b, miR-208 and miR-206) in CTR, *Zeb2-null* and *R26_Zeb2* mESCs, on day 22 from myogenic induction (**E**) or upon MyoD transfection (**D**). Each data point is represented as ΔC*_t_*, normalized for the housekeeping miR-16. Values are shown as mean ± SD; *n* = 3, * *p* < 0.05, ** *p* < 0.005, *** *p* < 0.0001.

The mESC lines used in all experiments are listed in material and methods sections [14,20]. Quantitative real-time polymerase chain reaction (qRT-PCR) analysis confirmed the absence of Zeb2 mRNAs in *Zeb2-null* mESCs, and increased mRNA in *R26_Zeb2* compared to controls (CTR) (Figure S1A). The expression of pluripotent markers (cMyc, Oct4, Sox2, Klf4, and Nanog) was confirmed in all mESC lines (Figure S1A) and lower levels of cMyc expression did not compromise the pluripotency of mESCs, as previously reported [21]. *Zeb2-null*, *R26_Zeb2* and control (CTR) mESCs were subjected to myogenic induction (Figure 1A), using the transient transfection of MyoD expression constructs [22,23]. The qRT-PCR analysis confirmed MyoD over-expression from transfected mESC lines (Figure 1B). At day 22, an up-regulation of myogenic markers (*Pax3*, *Pax7*, and *MyoD*) was found in *R26_Zeb2* compared to *Zeb2-null* and control samples, either in the absence or in the presence of MyoD transfection (CTR_MyoD_, *Zeb2-null_MyoD_*, and *R26_Zeb2_MyoD_*) (Figure 1C,D). *Myogenin* was upregulated only in *R26_Zeb2_MyoD_* compared to *Zeb2-null* and control samples (Figure 1D). In addition, the MyomiR (miR-1, miR-133b, miR-208, miR-206) expression profiles were altered in *Zeb2-null* and *R26_Zeb2* mESCs subjected to skeletal muscle differentiation. Interestingly, miR-1 and miR-133b, considered as markers of mesodermal transition and maturation [24], were up-regulated in *R26_Zeb2*, as compared to *Zeb2-null* and CTR both in the absence (Figure 1E) and in the presence of MyoD (Figure 1F). Furthermore, miR-206 which promotes myoblast differentiation [25], was similarly expressed in all samples in the absence of added MyoD (Figure 1E), whereas it was up-regulated in *R26_Zeb2_MyoD_*, as compared to *Zeb2-null_MyoD_* and CTR*_MyoD_* (Figure 1F). Then, miR-208 was down-regulated in *Zeb2-null*, as compared to *R26_Zeb2* and CTR in the absence of MyoD (Figure 1E). Similarly, miR-208 was highly expressed in *R26_Zeb2_MyoD_*, and it was totally absent in *Zeb2-null_MyoD_* and CTR_MyoD_ (Figure 1F). Consistent with the previous findings, the expression of myomiRs is strongly enhanced in *R26_Zeb2* mESCs, compared to CTR cells.

### 2.2. Zeb2 Enhances Skeletal Muscle Differentiation in Cultured Cells

**Figure 2:**
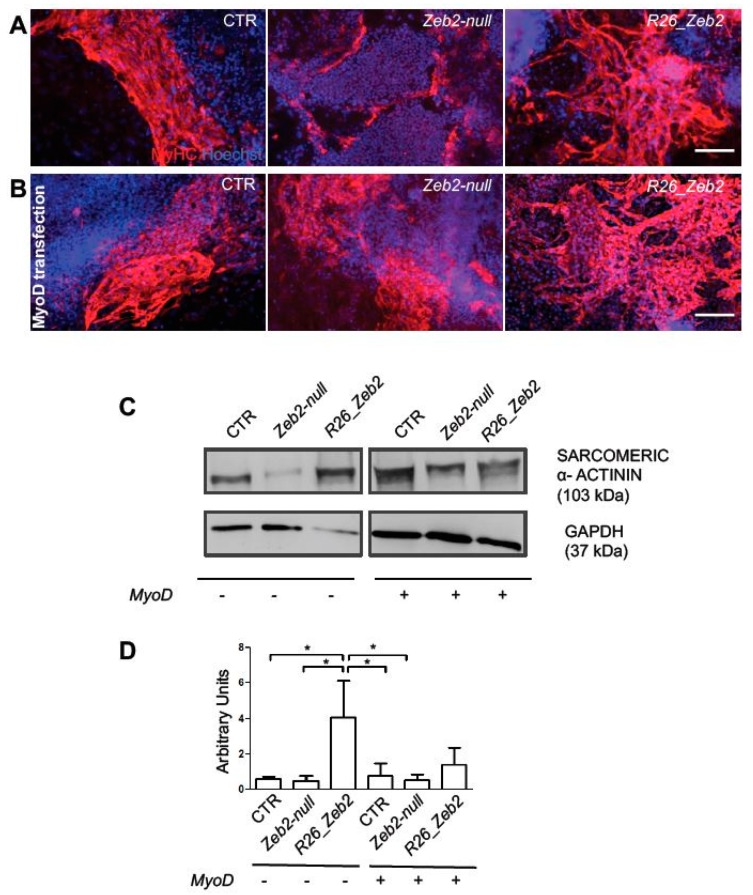
Protein localization and quantification of myogenic markers in *Zeb2-null* and *R26_Zeb2* mESCs subjected to myogenic differentiation. Immunofluorescence analysis for MyHC (red) after 22 days from myogenic induction (**A**) or upon MyoD transfection (**B**). The nuclei were stained in blue with Hoechst. Scale bars = 50 µm. (**C**) Example of WB analysis for sarcomeric α-actinin and GAPDH on day 22 from myogenic induction or upon MyoD transfection. (**D**) The quantification of WB analysis showed in C and normalized by the GAPDH housekeeping. Values are shown as mean ± SD; *n* = 3, * *p* < 0.05.

Consistent with these results, immunofluorescence analysis for myosin heavy chain (MyHC) (Figure 2A,B), and Western Blot (WB) analysis for sarcomeric α-actinin (Figure 2C, left panel) showed a marked increase of late myogenic markers in *R26_Zeb2*, compared to CTR and *Zeb2-null* samples in absence of MyoD. However, the forced expression of MyoD overcame the observed differences in skeletal muscle differentiation among samples (Figure 2C, right panels). These results suggest that *R26_Zeb2* mESCs exhibit a stronger myogenic commitment, compared to *Zeb2-null* and CTR samples (see also [14]). Next, we analyzed the effect of Zeb2 over-expression in C2C12 cell lines (Figure S2A,D). After 7 days of myogenic induction, Zeb2 mRNA levels remained higher in transfected cells as compared to controls (Figure S2A, left panel). Id3, a well known negative regulator of myogenesis, was upregulated in the transfected C2C12 cells and, strikingly, ZnfZeb2 exerted a stronger effect comparedto Zeb2 (Figure S2A, right panel). Myf5, MyoD and Myogenin were also up-regulated in C2C12 cells transfected with Zeb2 compared to control (Supplementary Figure S2B). However, in the C2C12 cells transfected with ZnfZeb2, Myf5, MyoD, Myogenin were down-regulated as compared to C2C12 cells transfected with Zeb2 (Figure S2B). Only the Pax3 was slightly upregulated in the C2C12 cells transfected with ZnfZeb2 as compared to C2C12 cells transfected with Zeb2 (and compared to control) (Figure S2B). Pax7 does not change the expression among control C2C12 cells transfected with Zeb2 or with ZnFZeb2 mutant (Figure S2B). Finally the myogenic differentiation potential of Zeb2 was confirmed by immunofluorescence and WB analyses (Figure S2C–F). In conclusion, consistent with the results obtained from pluripotent stem cells, the over-production of Zeb2 that is capable of binding to DNA enhanced myogenic differentiation in C2C12 cells.

### 2.3. Fluorescence-activated Cell Sorting-isolated CTR, Zeb2-null and R26_Zeb2 mCherry/MyoD-positive Cells and Single-cell RNA-sequencing 

**Figure 3:**
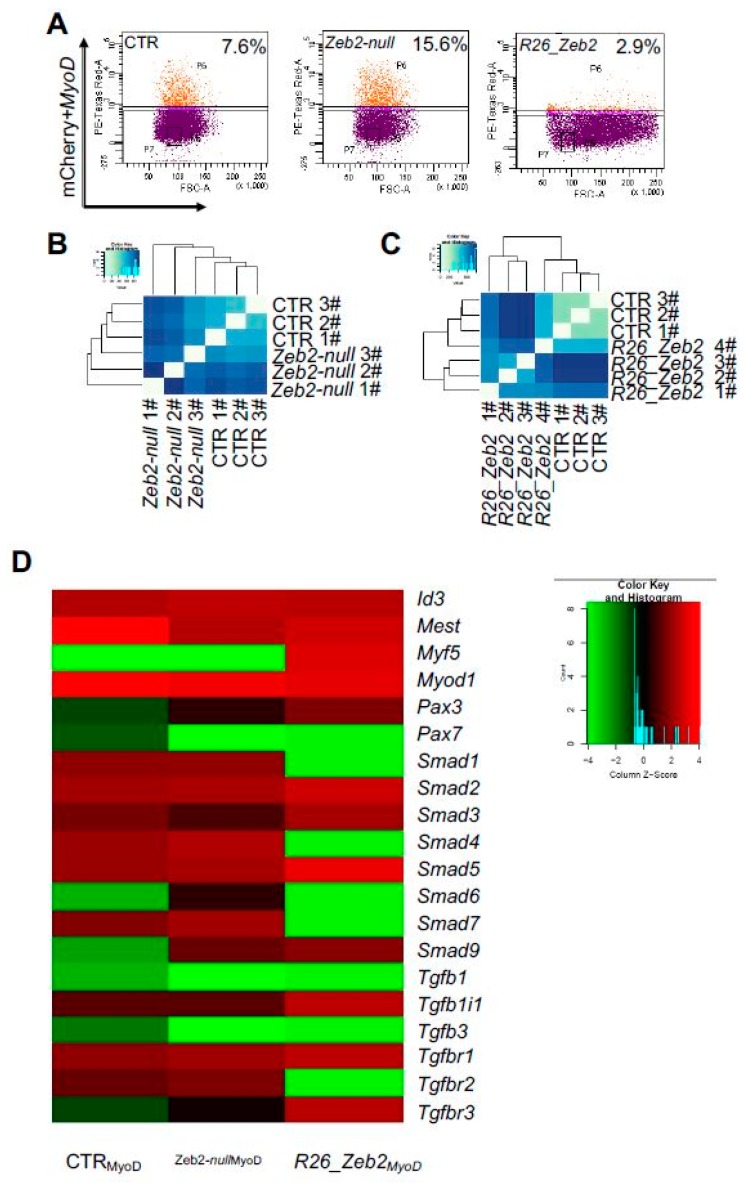
FACS-sorting and single cell RNAseq analysis of *Zeb2-null* and *R_26 Zeb2* mESCs. (**A**) Quantification of mCherry/MyoD-positive cells by fluorescence-activated cell sorting. Unbiased clustering of CTR mESCs vs *Zeb2-null* (**B**) and *R26_Zeb2* (**C**) mESCs analyzed by single RNA-seq. (**D**) The heatmap of expression levels of 20 myogenic regulatory genes from CTRL_MyoD_ (*n* = 3), *Zeb2-null*_MyoD_ (*n* = 3), and *R26_Zeb2*_MyoD_ (*n* = 4) sorted cells analyzed by single RNA-seq.

Sorted CTR, *Zeb2-null* and *R26_Zeb2* mCherry/MyoD-positive cells (Figure 3A) were then sorted as single-cells and submitted to RNA-sequencing. When clustered on a heatmap, patterns of single-cell gene expression emerged reflecting the origin of and similarity between the respective cell types (Figure 3B,C). However, upon MyoD transfection myogenic regulatory factors (MRFs), Id family, and SMAD/TGF-β pathway were altered in *Zeb2-null*_MyoD_ and *R26_Zeb2*_MyoD_ samples compared to CTR_MyoD_ (Figure 3D). Consistently, MyoD was similarly expressed in CTR_MyoD_, *Zeb2-null_MyoD_*, and *R26_Zeb2_MyoD_* (Figure 3D). Interestingly, *Id3* was slightly up-regulated in *Zeb2-null_MyoD_*, as compared to CTR*_MyoD_* and *R26_Zeb2_MyoD_*. *Mest* was highly expressed in CTR_MyoD_, but less in *R26_Zeb2*_MyoD_, as compared to *Zeb2-null_MyoD_*. *Myf5* was up-regulated in *R26_Zeb2*_MyoD_, as compared to *Zeb2-null_MyoD_* and CTR*_MyoD_* cells. Strikingly, we found *Smads6/7*, together with *Smad1* and *Smad4*, down-regulated in *R26_Zeb2*_MyoD_, compared to CTR and *Zeb2-null_MyoD_*. Moreover, *Smad2/3* and *Smad5* were up-regulated in *R26_Zeb2*_MyoD_, as compared to *Zeb2-null* and CTR. The top 30 highest expressed genes among CTR, *Zeb2-null*, and *R26_Zeb2* samples are reported (Figure S3A–C). Interestingly, we found highly expressed genes in *R26_Zeb2* such as *Malat1* (metastasis-associated lung adenocarcinoma transcript 1) and *Sparc* (secreted acidic cysteine rich glycoprotein), were both involved in skeletal muscle differentiation [26]. These results support the implication of Zeb2 in promoting myogenesis in pluripotent stem cells by modulating specific genes encoding components of the TGFβ/BMP signaling system.

### 2.4. GFP+ R26_Zeb2 Progenitor Cells Contiribute to Adult Myogenesis

**Figure 4:**
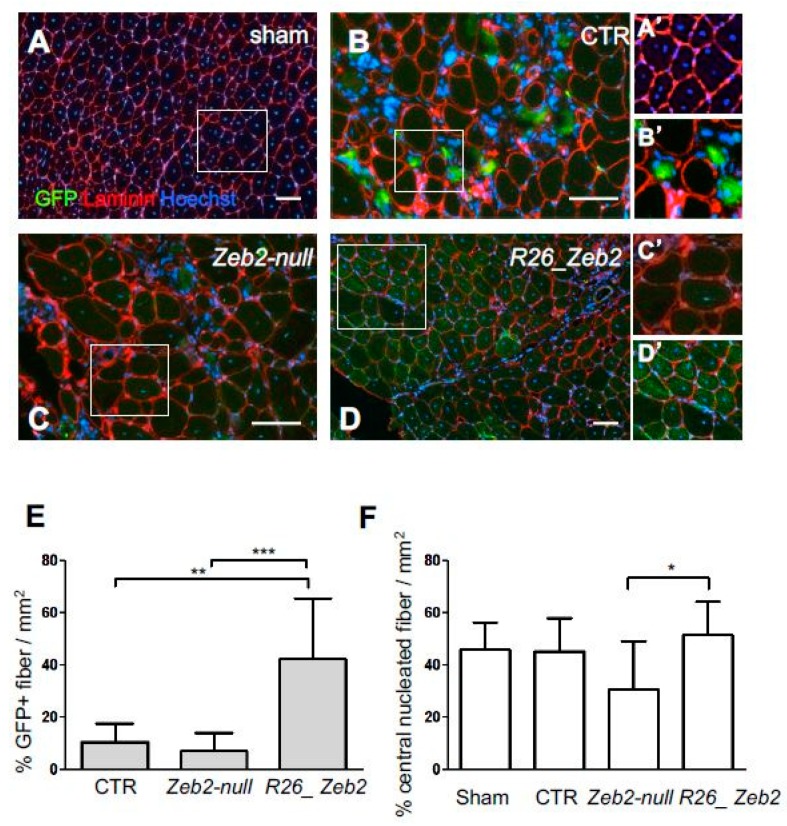
Intramuscular injections of *Zeb2-null* and *R26_Zeb2* mESC derivatives in an acute muscle injury model. Immunofluorescence analysis of GFP (green) and laminin (red) in sham-operated (**A**,**A**’) muscles or injected with CTR (*Zeb2 ^flox/flox^* as control) (**B**,**B**’), *Zeb2-null* (**C**,**C**’), and *R26_Zeb2* (**D**,**D**’) GFP^+^ mESC derivatives. Nuclei were stained in blue with Hoechst; scale bars = 100 µm. Quantification of GFP^+^ (**E**) and GFP^+^ central nucleated (**F**) fibers in injected muscles and expressed as mean ± SD; *n* = 5 (10 randomly selected fields were examined per sample); * *p* < 0.05, ** *p* < 0.005, *** *p* < 0.0001.

**Figure 5:**
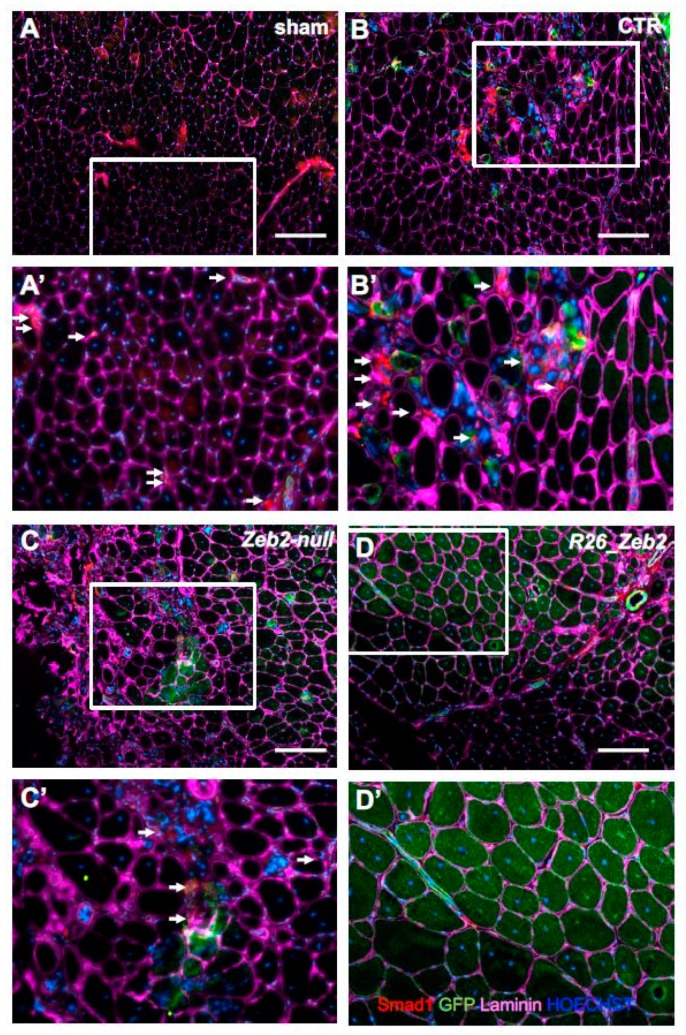
Smad1+ cells in muscles injected with *Zeb2-null* and *R26_Zeb2* mESC derivatives. Immunofluorescence analysis of Smad1 (red), GFP (green), and laminin (magenta) in sham-operated ((**A**) and inset (**A’**)) muscles, or injected with CTR (*Zeb2 ^flox/flox^* as control) ((**B**) and inset (**B’**)), *Zeb2-null* ((**C**) and inset (**C’**)), and *R26_Zeb2* ((**D**) and inset (**D’**)) GFP+ mESC derivatives. Arrows indicate Smad1+ nuclei. Nuclei were stained in blue with Hoechst; scale bars = 200 μm.

**Figure 6:**
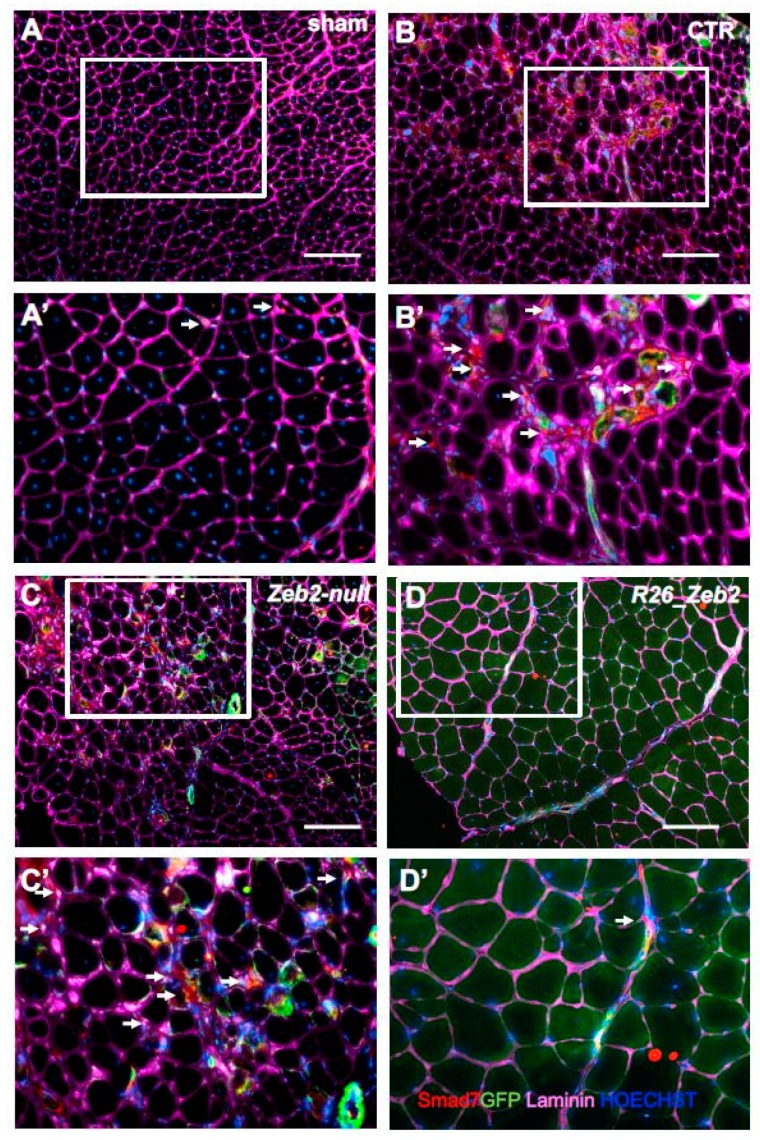
Smad7+ cells in muscles injected with *Zeb2-null* and *R26_Zeb2* mESC derivatives. Immunofluorescence analysis of Smad7 (red), GFP (green), and laminin (magenta) in sham-operated ((**A**) and inset (**A’**)) muscles, or injected with CTR (*Zeb2 flox/flox* as control) ((**B**) and inset (**B’**)), *Zeb2- null* ((**C**) and inset (**C’**)), and *R26_Zeb2* ((**D**) and inset (**D’**)) GFP+ mESC derivatives. Arrows indicate Smad7+. Nuclei were stained in blue with Hoechst; scale bars = 200 μm.

In order to assess the in vivo relevance of Zeb2 expression in skeletal muscle, the transgenic mESC derivatives, after myogenic induction, were injected into the CTX-treated *tibialis anterior* muscles of nude mice. In this acute model of muscle regeneration, the myogenic contribution of progenitors obtained from GFP^+^ CTR (GFP+ *Zeb2^flox/flox^*), GFP^+^ *Zeb2*-*null*, or GFP^+^ *R26_Zeb2* mESC derivatives were evaluated. After three weeks, the mice were sacrificed and, as expected, sham-operated muscles showed no GFP^+^ fibers (Figure 4A,A’). Interestingly, GFP^+^-*R26_Zeb2* progenitor cells integrated in regenerating *tibialis anterior* muscles (Figure 4D,D’) to a very large extent, compared to GFP^+^ CTR and GFP^+^ *Zeb2-null* cells (Figure 4B,C). GFP^+^ *Zeb2-null* progenitors were mainly localized in the interstitial space among the regenerating *tibialis anterior* muscles (Figure 4C,C’). The number of GFP^+^ fibers was significantly higher in *tibialis anterior* treated with the GFP^+^ *R26_Zeb2* progenitor cells, compared to muscles injected with GFP^+^ CTR or GFP^+^ *Zeb2*-*null* cells (Figure 4E). Moreover, the percentage of the central nucleated fibers of muscles treated with the GFP^+^ *Zeb2*-*null* cells (Figure 4F) was lower, as compared to GFP^+^ *R26_Zeb2* treated samples (Figure 4C), and the muscle structure of the former was highly disorganized. In order to validate the data extrapolated from the RNA sequencing analysis, we reported a down-regulation of the two inhibitory Smad genes (Smads6/7), together with Smad1 and Smad4 in *R26_Zeb2*, compared to CTR and *Zeb2-null* cells. Immunofluorescent staining for the expression of Smad1 (red) (Figure 5) or Smad7 (red) (Figure 6) signals coming from, GFP+ cells (green) was evaluated in sham-operated (A,A’) muscles, or injected with CTR (*Zeb2 ^flox/flox^* as control) (B,B’), *Zeb2-null* (C,C’), and *R26_Zeb2* (D,D’) GFP+ mESC derivatives. Remarkably, we found that the number of Smad 1 and Smad 7 positive fibers nuclei was significantly lower in *tibialis anterior* muscle treated with the GFP+ R26_Zeb2 (Figure 5D,D’; Figure 6D,D’) progenitor cells, compared to muscles injected with GFP+ CTR (Figure 5B,B’; Figure 6B,B’) or GFP*+ Zeb2-null* cells (Figure 5C,C’; Figure 6C,C’). These results are in agreement with the data obtained from the single cell RNA sequencing analysis. Finally, the NADH staining of muscles injected with GFP^+^ *Zeb2*-*null* progenitor cells showed a different pattern compared to other samples (Figure S4A–C; Figure S5A–C). No differences were observed in muscles injected with GFP^+^ *R26_Zeb2* progenitor cells compared to controls. These results indicated that Zeb2 is an important regulator of skeletal muscle differentiation in vivo, and that its enforced production leads to promotion of myogenic differentiation.

## 3. Discussion

In the current study, we analyzed the role of Zeb2 in skeletal muscle differentiation in pluripotent stem cells and in myogenic progenitors. For this purpose, we employed *Zeb2* genetic inactivation in mESCs cells and its transgenic rescue via the introduction of Zeb2-cDNA in transgenic *R26_Zeb2* mESCs (an approach previously used in vivo [27,28] and mESCs [14]). Zeb2 belongs to the ZEB family transcription factors involved in post-implantation (mouse) embryogenesis, organogenesis, and adult cell maturation from progenitors in a number of systems, and tumor progression. Zeb1 over-production in C2C12 cells inhibits both myotube formation and the expression of specific differentiation factors, such as MyHC and Myogenin [29]. While Zeb1 has been proposed to synergize with Smad proteins in order to activate TGFβ/BMP target genes, Zeb2 has the opposite effect [6,30]. 

*Zeb2-null* mESCs were confirmed here (see also [14]) to retain their pluripotency. However, when subjected to myogenic induction, they displayed a myogenic differentiation defect. Most strikingly, the over-production of Zeb2 in *R26_Zeb2* mESCs further improves their myogenic differentiation ability. This was mainly due to an up-regulation of MRFs, such as *Pax3, Pax7, MyoD, Myogenin*, and MyomiRs (miR-1, miR-133b, miR-206, and miR-208) in *R26_Zeb2* mESCs compared to CTR and *Zeb2-null* cells. These results suggest a potential role of Zeb2 in regulating muscle differentiation at miRNA level and, in turn, at gene level. The process of skeletal muscle differentiation is governed at least in part by miR-1, miR-206, and miR-133, which regulate the expression of MRF-encoding genes such as *MyoD*, *Myogenin*, and *Mef2* [31]. Indeed, microRNAs are important regulators involved in the establishment and maintenance of skeletal muscle differentiation [22,32,33,34]. For example, it was demonstrated that the expression of both miR-1 and miR-133 was enriched in striated muscle, and the differentiated myoblasts showed an elevated expression of miR-1 and miR-133 [32]. 

In order to address the question of whether the myogenic propensity of *R26_Zeb2* is compared to CTR and whether *Zeb2-null* mESCs would impact the transcriptome expression, we used single-cell RNA-sequencing to decomplexify Zeb2-modulated cell populations. Remarkably, we found a down-regulation of the two inhibitory Smad genes (*Smads6/7*), together with *Smad1* and *Smad4* in *R26_Zeb2* compared to CTR and *Zeb2-null* cells. These results were confirmed by an IF assay on muscle slides, where the down-regulation of Smad1 and Smad7 were also visible by a reduction of expression of these two transcription factors in GFP+ nuclei expressing cells in *R26_Zeb2*, when compared to CTR and *Zeb2-null* injected muscles. Furthermore, we detected *Smad2/3* and *Smad5* up-regulation in *R26_Zeb2* mESCs compared to CTR and *Zeb2-null* cells. Interestingly, it has been shown that Zeb2 binds to both Smads2/3 and Smads1/5/8 subclasses in ligand-stimulated cells, but it was also proposed that some functions of Zeb2 may be Smad-independent as well, and hence underpin its multiple mechanisms of action [6]. 

Additionally, we evaluated in the acute model of muscle regeneration whether the modulation of Zeb2 levels in myogenic progenitors generated from mESCs would impact their myogenic differentiation potential in vivo. The results obtained in acute muscle injury model suggest that Zeb2 represents a promising target to exploit an improvement of skeletal muscle regeneration. We also used Zeb2 over-production in C2C12 mouse myoblasts, and confirmed that such Zeb2 modulation positively impacts the myogenic potential of adult muscle progenitors (see supplemental information). This occurs even when using a *ZnfZeb2* mutant that does not bind to DNA, indicating a squelching effect by such mutant protein on myogenic relevant partners of normal Zeb2, perhaps activated Smads in the nucleus in complex with Smad4 [7]. The enhanced skeletal muscle differentiation observed in Zeb2 over-producing C2C12 cells might be mediated directly by prevention of SMAD-action on Smad-modulated target genes. Indeed, we found that one of the *Id* genes, *Id3*, acknowledged BMP-Smad induced genes whose BMP-stimulated transcription is downregulated by Zeb2 (Conidi and Huylebroeck, *unpublished results*), and was strongly up-regulated in C2C12 transfected with a *ZnfZeb2* mutant. Previously, in perturbation studies (of *Smad1/5* and *8*, now named *9*), BMP-Smad signaling was shown to have impact on skeletal muscle differentiation in pluripotent stem cells and myogenic progenitor cells [35]. 

In conclusion, this study is pioneering for the functions of Zeb2 in skeletal muscle differentiation. Our results underline that Zeb2 has a crucial role in triggering myogenic differentiation. We also highlighted the complexity of the myogenic transcriptional regulation in myogenic commitment and differentiation, where bHLH transcription factors are also supported by zinc-finger proteins.

## 4. Materials and Methods

### 4.1. Cell Culture and in vitro Skeletal Muscle Differentiation of mESCs

The mESC lines were obtained by intercrossing *Zeb2 ^flox/flox^* CD1 mice [20]. *Zeb2*-null mESCs were obtained by transient transfection with Cre-recombinase carrying plasmid, while *R26_Zeb2* lines were generated inserting N-terminally flag-tagged, wild-type *Zeb2* cDNA into the Rosa26 locus of the *Zeb2-null* mESCs [14]. In all experiments, *Zeb2 ^flox/flox^* mESCs were used as control (CTR). The CTR, *Zeb2-null*, and *R26_Zeb2* mESCs were grown on a feeder layer of inactivated Murine Embryonic Fibroblasts (iMEFs), in knockout DMEM (Gibco-Life Technologies, Waltham, MA, USA) supplemented with 15% of Fetal Bovine Serum (FBS), 100 U/mL penicillin, and 100 μg/mL streptomycin, 200 nM glutamine, MEM non-essential amino acids, 0.2% 2-mercaptoethanol (Gibco-Life Technologies, Waltham, MA, USA), and 1000 U/mL of leukemia inhibitory factor (LIF, Merck Millipore, Burlington, MA, USA). For in vitro skeletal muscle differentiation, CTR, *Zeb2-null*, and *R26_Zeb2* mESCs were detached, and a feeder layer was removed after 30 min of incubation with fresh coated gelatin. After pre-plating, the mESCs were induced to form Embryoid Bodies (EBs) in six well plate ultra-low attachments (Corning, New York, NY, USA), in a growth medium without LIF for 2 days. Subsequently, the EBs were gently collected to avoid disruption, centrifuged, and plated in collagen-coated six well plate (Nunc, Thermo Fisher Scientific, Waltham, MA, USA) in EB medium containing Iscove’s modified Dulbecco’s medium (IMDM), supplemented with 15% FBS, 1% penicillin/streptomycin, 1% L-glutamine (all from Thermo Fisher Scientific, Waltham, MA, USA), 4.5 mM monothioglycerol (Sigma-Aldrich, Milan, Italy), 50 μg/mL ascorbic acid (Sigma-Aldrich, St. Louis, MO, USA), and 200 μg/mL iron-saturated transferrin (Sigma-Aldrich, St. Louis, MO, USA). 

In parallel, CTR, *Zeb2-null* and *R26_Zeb2* EBs were transfected with MyoD. The EBs were disrupted by pipetting, and transfected in suspension with non-integrating plasmid MyoD transcripts under a CMV promoter (PLV CMV MyoD), using Effectene transfection reagent (Qiagen, Hilden, Germany), according to the manufacturer’s protocol.

To induce terminal differentiation, at day 11, EBs were incubated in DMEM with 2% heat-inactivated horse serum, 1% glutamine, and 1% penicillin-streptomycin (all from Thermo Fisher Scientific, Waltham, MA, USA).

In order to perform the in vivo experiment, CTR, *Zeb2-null*, and *R26_Zeb2* mESCs were transduced with GFP carrying lentivirus. Transductions were carried out within 48 h of virus incubation on 6 well plates (Corning, New York, NY, USA). After sorting, the GFP^+^ mESCs were cultured on a feeder layer. 

For in vivo skeletal muscle differentiation experiments, the GFP^+^ CTR, *Zeb2-null* and *R26_Zeb2* progenitor cells after the induction to form EBs were used. After 2 days, the EBs were collected and plated on collagen coated six wells plates (Corning). After 7 days in the IMDM medium, the GFP^+^ CTR, *Zeb2-null*, and *R26_Zeb2* progenitor cells were detached and injected in nude mice.

The C2C12 cell line was maintained in Dulbecco’s Eagle’s medium (DMEM) high glucose supplemented with 10% FBS, 2 mM glutamine, 100 U/mL penicillin, 100 μg/ml streptomycin, and 1 mM of sodium pyruvate (all from Thermo Fisher Scientific, Waltham, MA, USA). The myogenic differentiation was induced by changing the medium to DMEM high glucose supplemented with 2% of heat-inactivated horse serum, 1% glutamine, and 1% penicillin-streptomycin (all from Thermo Fisher Scientific, Waltham, MA, USA).

### 4.2. RNA Extraction and Quantitative Real-Time PCR Analysis

RNA was extracted with Purelink RNA Mini Kits (Invitrogen, Life Technologies, Waltham, MA, USA), and genomic DNA traces were removed with Turbo DNase (Invitrogen, Life Technologies, Waltham, MA, USA), following the manufacturer’s instructions. A qRT-PCR was performed on mESCs as described in [34]. The values represent the mean ± SD of three independent experiments performed in triplicate. Glyceraldehyde 3-phosphate dehydrogenase, (GAPDH) was used as the housekeeping gene, to normalise the mRNA levels, and the data are shown as ΔCt (Ct_gene of interest_ − Ct_GAPDH_). Primers: Zeb2-Fw: CAATGCAGCACTTAGGTGGTA, Zeb2-Rev: TTGCCTAGAAACCGTATTGT; Oct4-Fw: CCAGGCAGGAGCACGAGTGG, Oct4-Rev: CCACGTCGGCCTGGGTGTAC; Sox2-Fw: GAGTGGAAACTTTTGTCCGAGA, Sox2-Rev: GAAGCGTGTACTTATCCTTCTTCAT; Klf4-Fw: GGCGAGTCTGACATGGCTG, Klf4-Rev: GCTGGACGCAGTGTCTTCTC; Nanog-Fw: CAGGTGTTTGAGGGTAGCTC, Nanog-Rev: CGGTTCATCATGGTACAGTC; cMyc-Fw: GCTCCATCCACTCCCTTTAC, cMyc-Rev: CCCTCCAGATCAGTTCCTTTATC; Pax3-Fw: TGCGTCTCTAAGATCCTGTGCAG, Pax3-Rev: CAGCTGCTCTGCCGTGAAGGTGGT; Pax7-Fw: CCCTCAGTGAGTTCGATTAGCC, Pax7-Rev GGTCGGGTTCTGATTCCACA; MyoD-Fw: GAGCAAAGTGAATGAGGCCTT, MyoD-Rev: CACTGTAGTAGGCGGTGTCGT; Myogenin-Fw: ATGGAGCTGTATGAGACATCCCC, Myogenin-Rev: CGACACAGACTTCCTCTTACAC, ID3-Fw: TGCTACGAGGCGGTGTGCTG, ID3-Rev: TGTCGTCCAAGAGGCTAAGAGGCT; Gapdh-Fw: GGAAGCCCATCACCATCTT; Gapdh-Rev: GGTGGTAAAGACACCAGTAGAC.

### 4.3. miRNA Isolation and Quantification

Small RNA extractions from CTR, *Zeb2-null*, and *R26_Zeb2* mESCs, after 22 day of skeletal muscle differentiation, were performed using PureLink miRNA Isolation kits (Invitrogen, Life Technologies, Waltham, MA, USA), following the manufacturer instructions [34]. The relative quantification of the miRNA targets was carried out using the ΔCt formula (Ct_miRNA of interest_ - Ct_miR-16_), according to the Ct method. Three independent experiments were performed.

### 4.4. Immunofluorescence Analysis

Tissue slides (7 µm) and cells were fixed with 4% of paraformaldehyde (PFA, Polysciences Polysciences, Valley Road Warrington, USA) in Phosphate Buffer Saline (PBS), permeabilized with 0.2% Triton X-100 in PBS containing 1% (w/v) of bovine serum albumin, and blocked with 1:10 donkey serum (all from Sigma-Aldrich, St. Louis, MO, USA). Primary antibodies: 1:500 goat anti-GFP (Abcam, Cambridge, United Kingdom), 1:300 rabbit anti-laminin (Sigma-Aldrich, St. Louis, MO, USA), 1:3 mouse anti-MyHC (DSHB, clone MF20, Developmental Studies Hybridoma Bank, Iowa City, Iowa, USA), 1:50 mouse anti Smad1 (LSBio, Seattle, Washington, (CTX, Naja Mossambica, Sigma-Aldrich, St. Louis, MO, United States, USA) USA) and 1:100 mouse Smad7 (R&D Systems, Inc., Minneapolis, USA) were incubated overnight in PBS. The secondary Alexa Fluor donkey antibodies (Invitrogen, Life Technologies, Waltham, MA, USA) were diluted 1:500. Nuclei were counterstained with 1:10,000 Hoechst (Sigma-Aldrich, St. Louis, MO, USA).

### 4.5. Western Blotting

The WB analysis was performed on 40 µg of protein lysates from undifferentiated and differentiated mESCs and the C2C12 myoblast cell lines in Ripa buffer were supplemented with 10 mM of Sodium Fluoride, 0.5 mM Sodium Orthovanadate, 1:100 Protease Inhibitor Cocktail (Sigma), and 1 mM Phenylmethylsulfonyl Fluoride (PMSF) (all from Sigma-Aldrich, St. Louis, MO, USA). Primary antibodies: rabbit anti-sarcomeric α-actinin (Abcam, Cambridge, United Kingdom) 1:500; mouse anti-MyHC (DSHB, clone MF20, Developmental Studies Hybridoma Bank Iowa City, Iowa, USA) 1:3; mouse anti-GAPDH (Sigma-Aldrich, St. Louis, MO, USA) 1:1000; mouse anti-tubulin, beta (Merck Millipore, Burlington, MA, USA) 1:1000. The secondary antibodies were HRP-conjugated antibodies (Santa Cruz Biotechnology, Dallas, Texas, USA)1:5000. Bands were detected at GelDoc (Bio-Rad, Hercules, CA, USA), after the incubation with SuperSignal Pico chemiluminescent substrate (Thermo Fisher Scientific, Waltham, MA, USA).

### 4.6. FACS-isolation and Single cell RNA-Sequencing Analysis of CTR, Zeb2-null and R26_Zeb2 mESCs

At day 2, CTR, *Zeb2-null* and *R26_Zeb2* EBs were disrupted by pipetting, and transfected with the mCherry fluorescence reporter (mCherry-2A-PuR) [36] and MyoD plasmids (1:3 ratio) by the Effectene transfection reagent (Qiagen, Hilden, Germany). Two days later, EBs were disrupted and sorted for the mCherry fluorescence reporter. Manual single-cell isolation was performed in CTR, *Zeb2-null*, and *R26_Zeb2* mCherry/MyoD-positive cells from PBS suspensions using a STRIPPER micropipetter (Origio, CooperCompanies, San Ramon, CA, USA), and a 135 µm tip. Each CTR, *Zeb2-null*, and *R26_Zeb2* mCherry/MyoD-positive single cell was transferred into a lysis buffer and processed in accordance to protocol [37]. Tagmentation was carried out by using the Illumina Nextera XT DNA Sample Preparation Kit (Illumina, San Diego, CA, USA). 

### 4.7. RNA Sequencing and Bioinformatics Analysis

Each library was sequenced on an Illumina HiSeq2500 sequencer (San Diego, CA, USA), according to the manufacturer’s recommendations, generating 50 bp single-end reads. Between 3 and 21 million reads per sample were produced. Quality control of raw reads was performed with FastQC v0.11.5. Adapters were filtered with ea-utils v1.2.2.18. Splice-aware alignment was performed with TopHat v2 against the mouse reference genome mm10. The number of allowed mismatches was 2. Reads that mapped to more than one site to the reference genome were discarded. The minimal score of alignment quality to be included in count analysis was 10. Resulting SAM and BAM alignment files were handled with Samtools v0.1.19.24. Quantification of reads per gene was performed with HT-Seq count v0.5.3p3, using Ensembl annotation version 78. Count-based differential expression analysis was done with R-based Bioconductor package DESeq [38]. The data discussed in this publication have been deposited in NCBI’s Gene Expression Omnibus, and are accessible through GEO Series accession number GSE147744.

### 4.8. In vivo Experiments

All animal procedures were performed according to the guidelines of the Animal Welfare Committee of KU Leuven and Belgian/European legislation. NMRI-nu (Rj:NMRI-Foxn1nu/Foxn1nu)Intramuscular injections of 2 × 10^5^ GFP^+^CTR, *Zeb2-null*, and *R26_Zeb2* progenitor cells in vitro with the IMDM medium after the formation of EBs were performed in 4-week-old male athymic nude mice (NMRI-nu (Rj:NMRI-Foxn1nu/Foxn1nu, Charles River; *n* = 5 for each group). Mice received a 10µM solution of Cardiotoxin (CTX, Naja Mossambica, Sigma-Aldrich, St. Louis, MO, USA) to induce muscle damage in the *Tibialis Anterior* (TA), *Gastrocnemius* (GSN), and *Quadriceps* (QD) of each hind limb at 48 h before cell injection. After three weeks, the mice were sacrificed by cervical dislocation, and TA, GSN, and QD were excised and stored at −80 °C for further analysis. The TA was processed for histological immunofluorescence analysis. The sham-operated muscles were used as a negative control where the mice received equal treatment, as well as amounts of cell-free saline solution. Engraftment and/or regeneration outcome were investigated 3 weeks after injection. To quantify both the GFP^+^ fibers and the central nucleated fibers in each coverslip/sample was calculated using the ImageJ software.

### 4.9. Statistical Analysis

The statistical significance among multiple samples was determined with the one-way ANOVA analysis of variance with Bonferroni’s multiple comparison tests. When two groups were compared, a non-parametric *t*-test was used. The data are shown as the mean ± SD as indicated. All data were acquired from at least three independent experiments. All statistical tests were performed via Prism5 GraphPad software (Abacus Concepts, GraphPad Software, SanDiego, CA, USA). 

## Supplementary Materials

Supplementary materials can be found at https://www.mdpi.com/1422-0067/21/7/2525/s1.
