# Supplementary material for: Zeb2 Regulates Myogenic Differentiation in Pluripotent Stem Cells
Source: Int J Mol Sci. 2020 Apr 5;21(7):2525. doi: 10.3390/ijms21072525 (PMC7177401; doi:10.3390/ijms21072525)

Zeb2 Regulates Myogenic Differentiation in Pluripotent Stem Cells

Ester Sara Di Filippo[1,2], Domiziana Costamagna[2], Giorgia Giacomazzi[2], Álvaro Cortés-Calabuig[3], Agata Stryjewska[2,+], Danny Huylebroeck[2,4], Stefania Fulle[1] and Maurilio Sampaolesi[2,5,*]

[1] Dept. of Neuroscience Imaging and Clinical Sciences, University "G. d'Annunzio" of Chieti-Pescara, 66100 Chieti, Italy;
[2] Dept. of Development and Regeneration, KU Leuven, 3000 Leuven, Belgium;
[3] Laboratory for Cytogenetics and Genome Research, KU Leuven, 3000 Leuven, Belgium;
[4] Department of Cell Biology, Erasmus University Medical Center, 3015 CN Rotterdam, The Netherlands;
[5] Human Anatomy Unit, Department of Public Health, Experimental and Forensic Medicine, University of Pavia, 27100, Pavia, Italy;
[$] *Present address*: Neural Development, Plasticity and Repair, Wolfson Institute for Biomedical Research, University College London, London WC1E 6BT, U.K.
* Correspondence: maurilio.sampaolesi@kuleuven.be

SUPPLEMENTAL INFORMATION

Supplementary Materials and Methods

NADH – transferase staining.

Muscle sections (7 µm) were rehydrated into PBS and incubated for 20 minutes at 37 °C with 0,4 mg/ml of NADH, 0,8 mg/ml of NBT in 0,1M Tris HCl. Washed twice in water, dehydrated in 75% of Ethanol for 1 minute, 95% of Ethanol for 1 minute 100% of Ethanol for 5 minutes. Remove the Ethanol with Xylene for 5 minutes. The day after, the sections were mounted with glycerol and then photographed. To quantify both the glycolytic fibers and the oxidative fibers in each coverslip/sample was calculated using the ImageJ software.

Plasmids and transfection assays in C2C12 myoblast cell lines. Transfection experiments with Zeb2 and *ZnfZeb2* mutant plasmids were carried out using Lipofectamine 2000 (Invitrogen). The day before the transfection, the C2C12 cells were seeded according to the manufacturer's protocol. The DNA-Lipofectamine complexes were incubated for 20 minutes at room temperature, added directly to the cell lines and after 9 h the growth medium was added. The following day, the growth medium was removed and was replaced with the differentiation medium.

Supplementary Figures

Figure S1

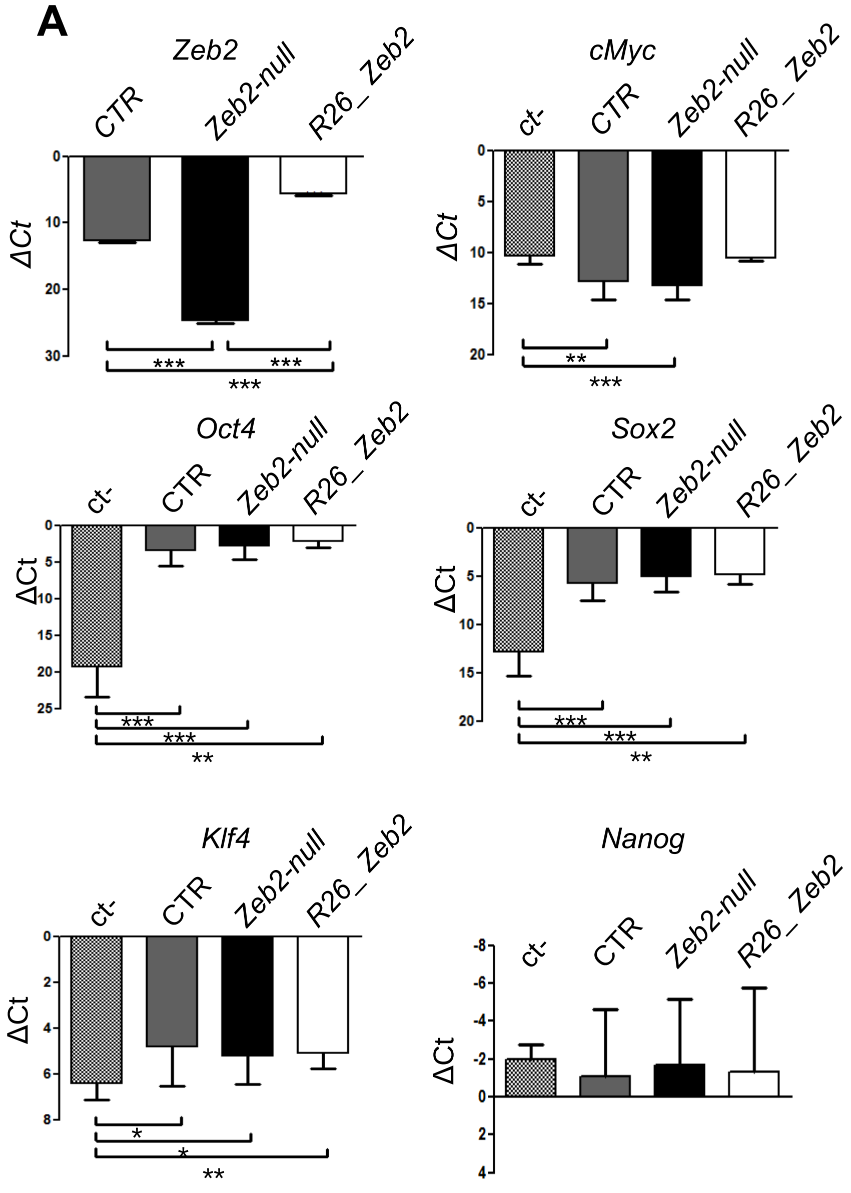

Figure S1. Pluripotent gene expression in *Zeb2-null* and *R26_Zeb2* mESCs.

(A) qRT–PCR analysis for the expression of *Zeb2, cMyc, Oct4, Sox2, Klf4* and *Nanog* in wt (CTR), *Zeb2-null* and *R26_Zeb2* mESCs and in MEF (ct-). Values are shown as mean ± SD, n= 3, *p<0.05, **p<0.005, ***p<0.0001.

Figure S2

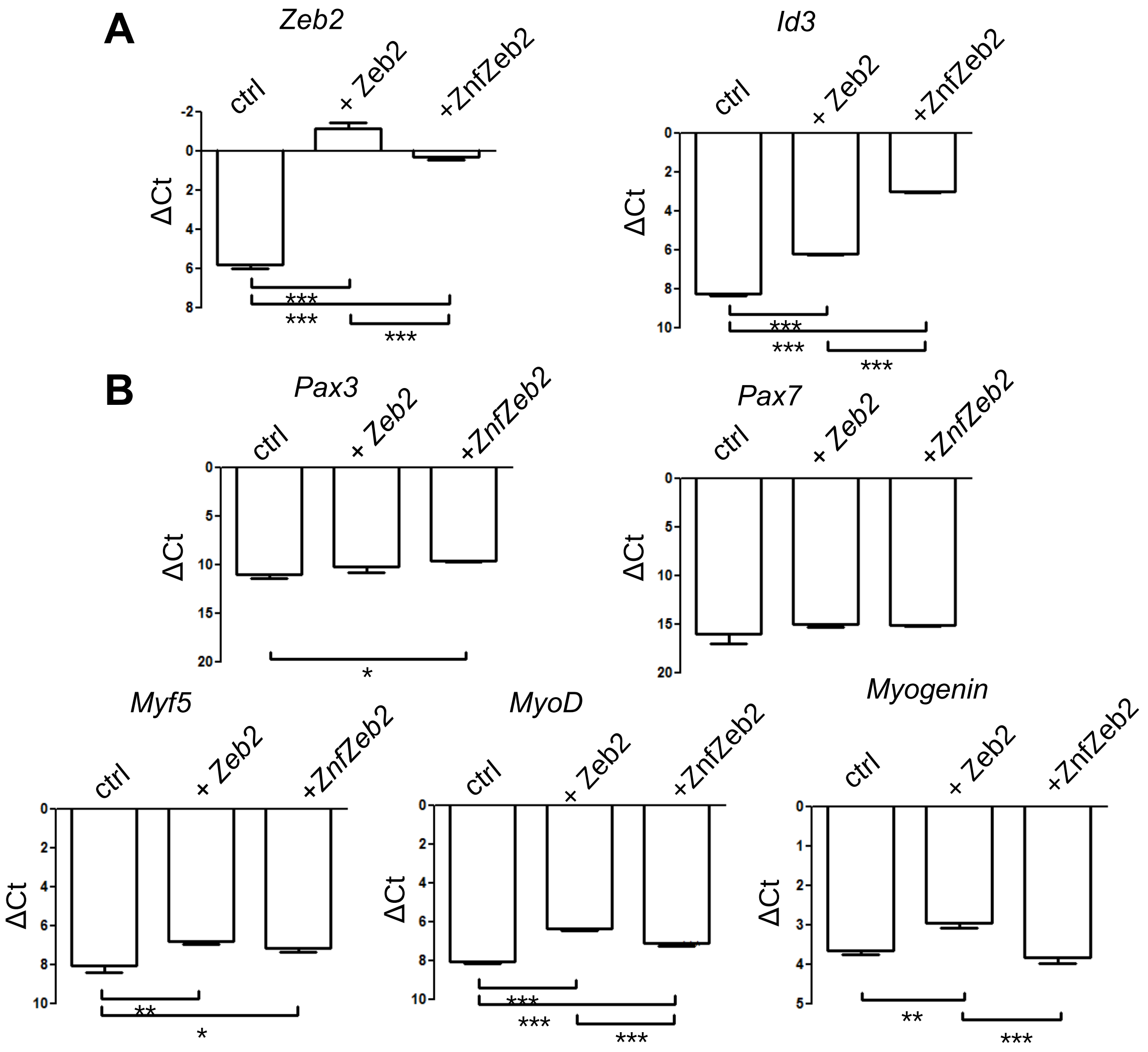

Figure S2

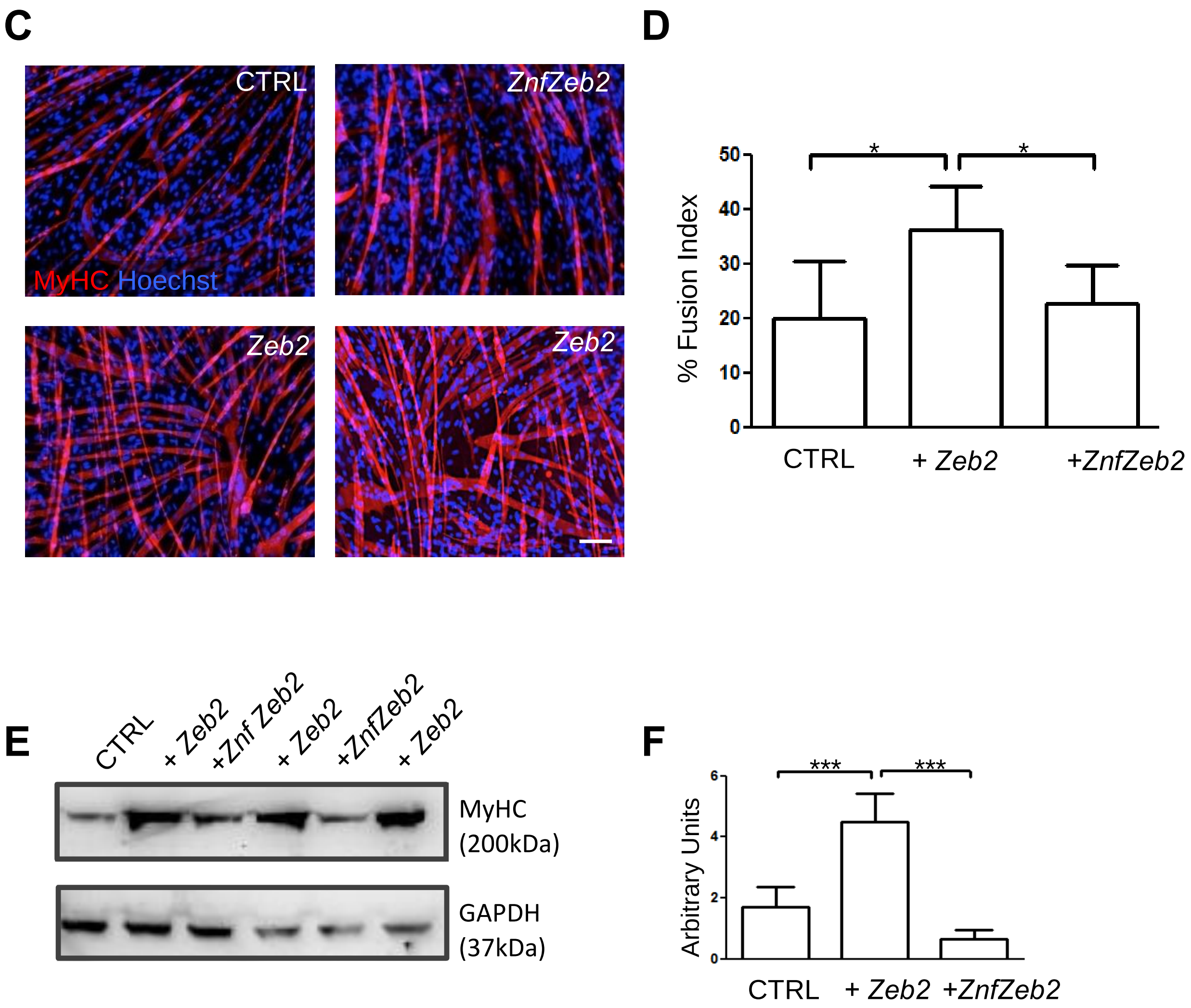

Figure S2. Zeb2 overexpression in C2C12 cells.
(A) C2C12 were transfected with Zeb2 (+ Zeb2) or with *ZnfZeb2* mutant (+*ZnfZeb2*). At day 7 from serum starvation when C2C12 were fully differentiated, qRT-PCR analysis for the Zeb2 and Id3 mRNA levels was performed. (B) qRT-PCR analysis for Pax3, Pax7, Myf5, MyoD and Myogenin at day 7 from myogenic induction in C2C12 transfected with Zeb2 (+Zeb2) or *ZnfZeb2* mutant (+*ZnfZeb2*) plasmids. Gapdh was used as a housekeeping gene for normalization. Values are shown as mean ± SD; n = 3, *p<0.05, **p<0.005, ***p<0.0001. (C) Immunofluorescence analysis of MyHC (in red) at day 7 from myogenic induction in C2C12 transfected with Zeb2 (+Zeb2) or *ZnfZeb2* mutant (+*ZnfZeb2*) plasmids. Nuclei were stained in blue with Hoechst. Scale bars = 50 μm. (D) The percentage of fusion index in C2C12

transfected with Zeb2 (+Zeb2) or *ZnfZeb2* mutant (+*ZnfZeb2*) plasmids are expressed as mean ± SD; n = 5 (10 randomly selected fields were examined per sample); *p<0.05. (E) Example of WB analysis for MyHC and GAPDH in samples showed in C. (F) Quantification of WB analysis shown in E. Values are shown as mean ± SD; n = 3, ***p<0.0001

Figure S3

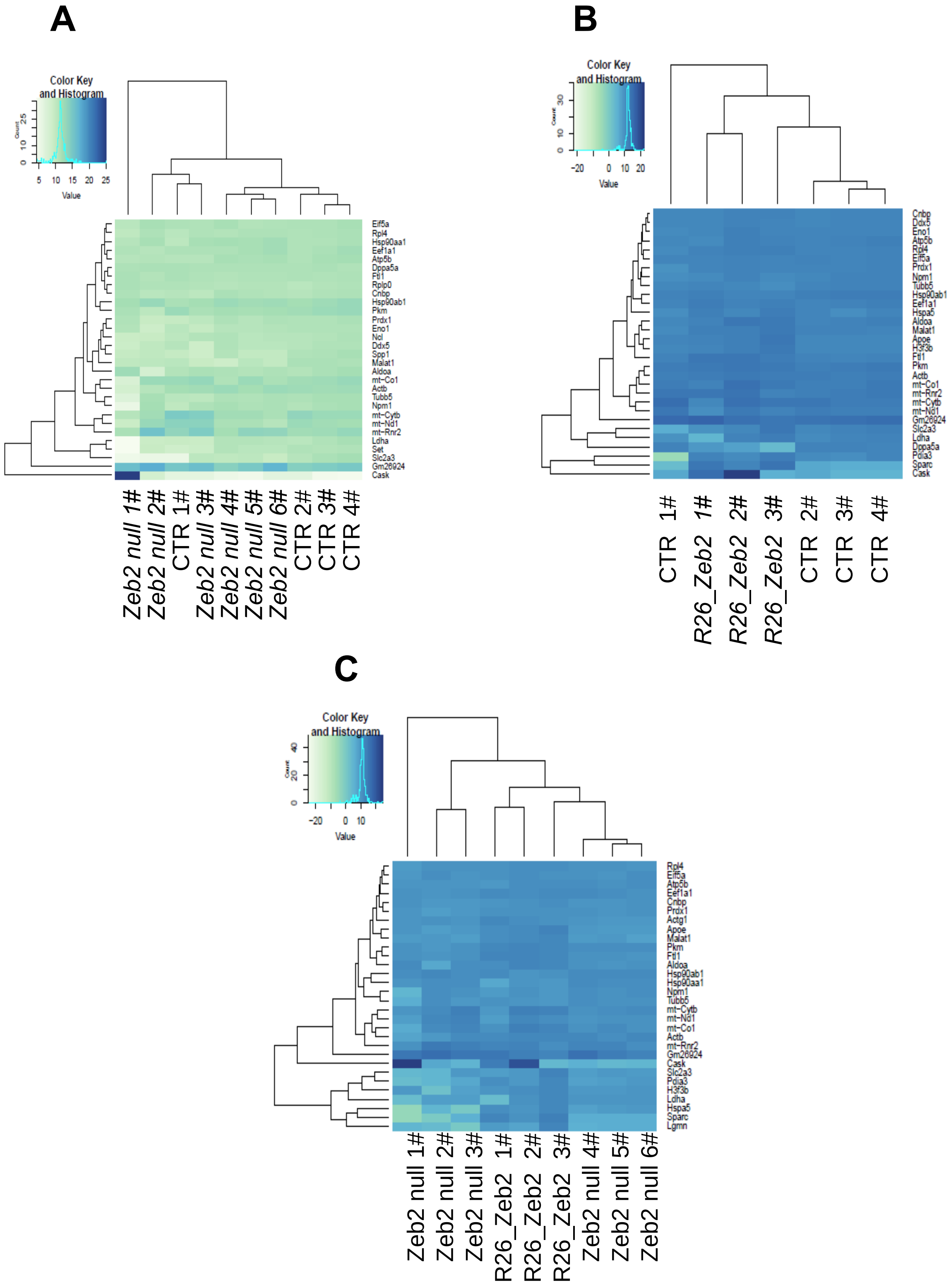

Figure S3. A heatmap of the top 30 highly expressed genes in wt (CTR), *Zeb2-null* and *R26_Zeb2* mCherry/MyoD-positive cells.
(A) Comparison between CTRL (n=4) and *Zeb2-null* (n=6) single cells. (B) Comparison between CTR (n=4) and *R26_Zeb2,* (n=3) single cells. (C) Comparison between *Zeb2-null* (n=6) and *R26_Zeb2* (n = 3) single cells.

Figure S4

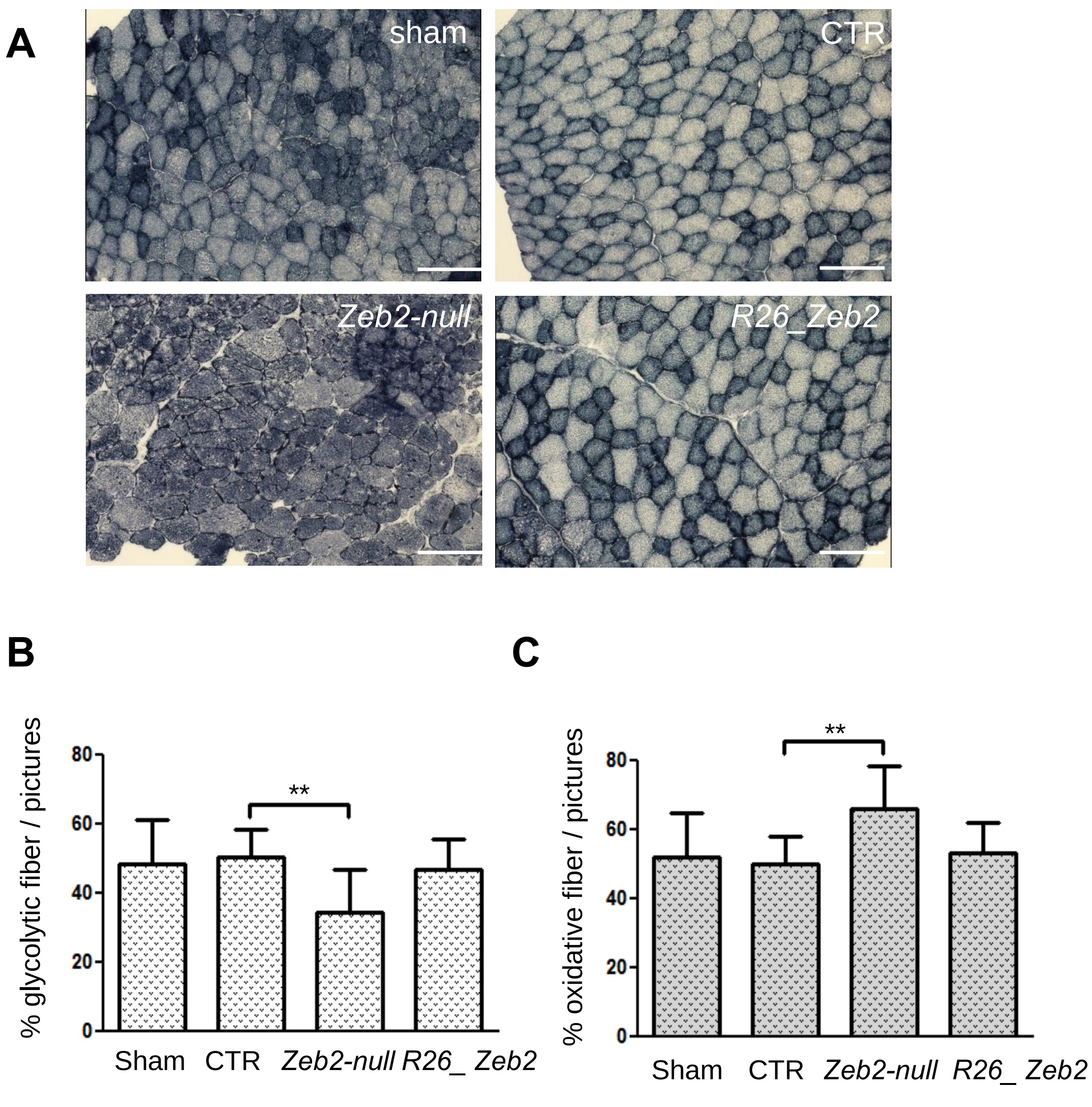

Figure S4. Oxidative and glycolytic muscle fibers in acute injured muscles transplanted with GFP+ wt, *Zeb2-null* and *R26_Zeb2* mESC derivatives.
(A) NADH – transferase staining in cross sections of *tibialis anterior* muscles from sham mice or treated with GFP+ CTR (*Zeb2flox/flox*), GFP+ Zeb2-null, or GFP+

R26_Zeb2 mESC derivatives. (B, C) Quantification of oxidative and glycolytic fiber types from *tibialis anterior* muscles of treated mice. Data are expressed in percentages as mean ± SD, n = 5 independent experiments (10 randomly selected fields per sample were examined). ** p<0,005.

Figure S5

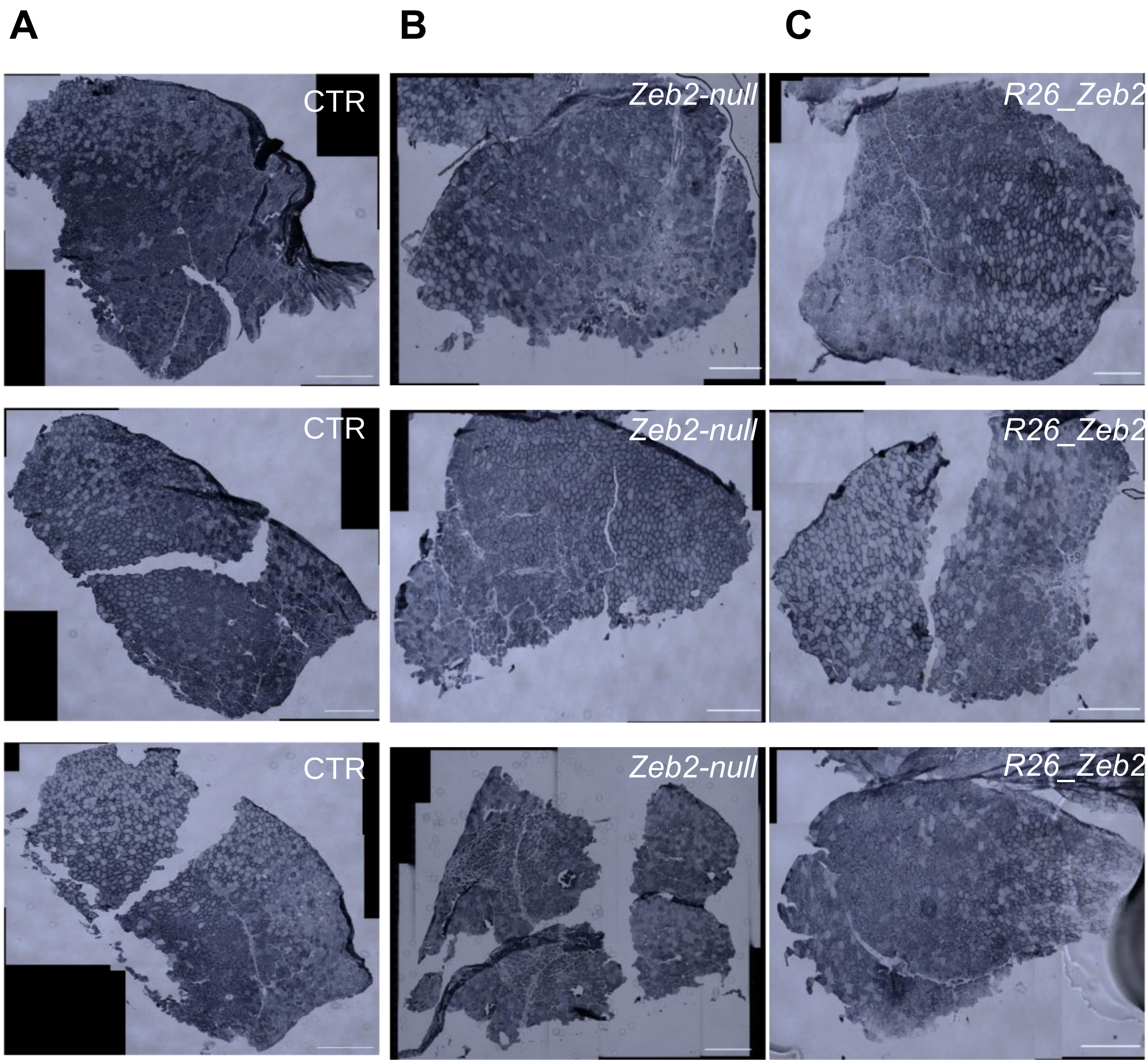

Figure S5. Examples of whole muscle section reconstructions upon NADH – transferase staining of treated muscles. NADH transferase staining of *tibialis anterior* cross-sections from muscles treated with GFP+ CTR (*Zeb2flox/flox*), GFP+ Zeb2-null, or GFP+ *R26_Zeb2* mESC derivatives. Scale bars = 200 μm.

Uncropped western blot filters used in the figures

WB filter used in Figure 2C

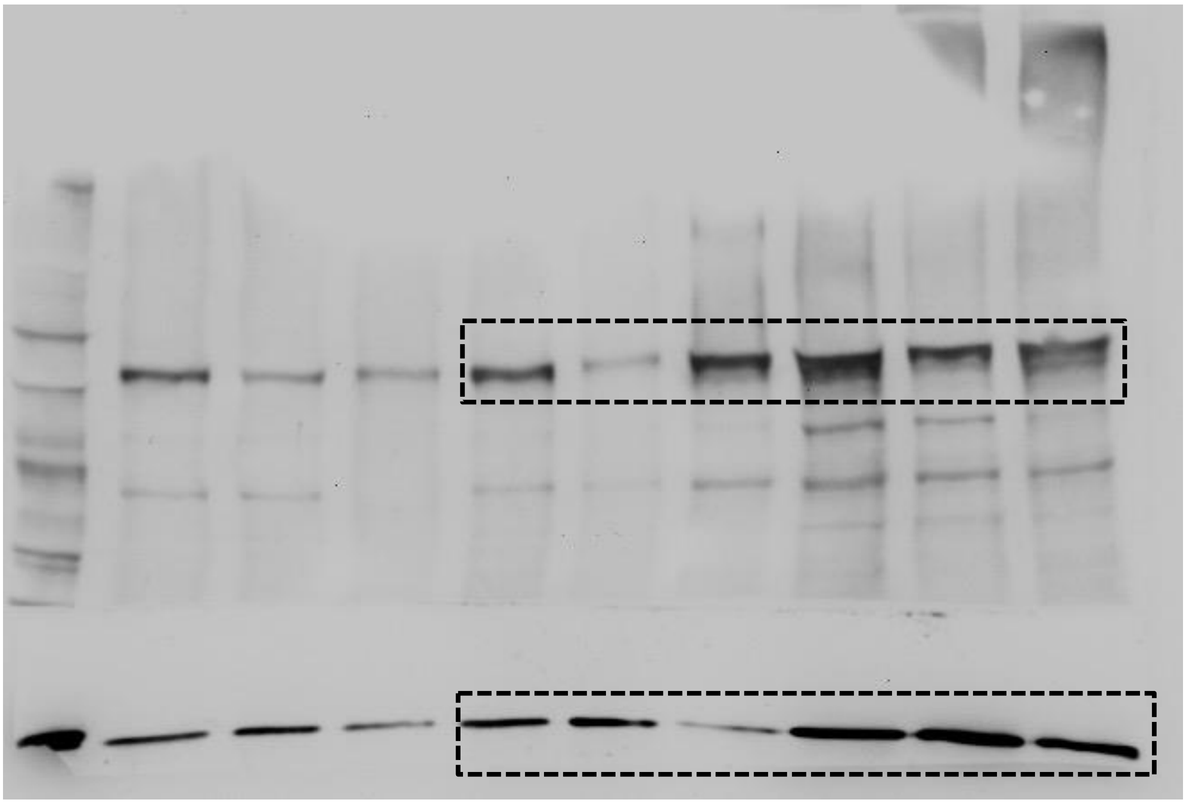

WB filters used in Figure S2E

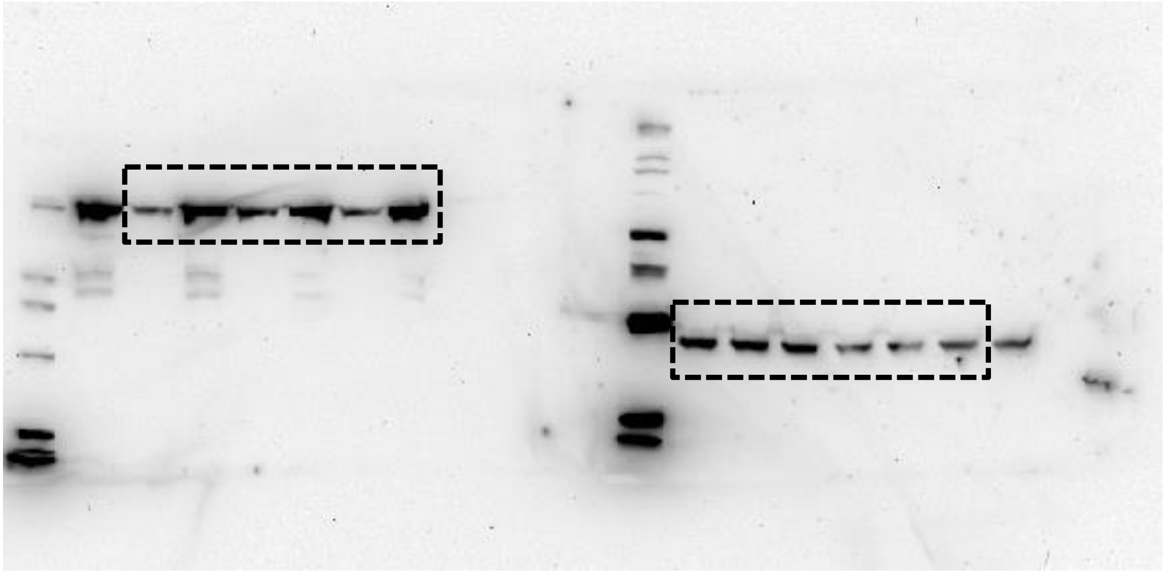

Supplement: Supplementary file 1 [file ijms-21-02525-s001.pdf]
